# Rethinking the Platinum Standard in SCLC: A Meta-Analysis of Limited- and Extensive-Stage Disease in the Immunotherapy Era

**DOI:** 10.1016/j.jtocrr.2026.101055

**Published:** 2026-07-24

**Authors:** Tomer Meirson, Hadas Ditzian Kugler, Ekaterina Hanovich, Ofer Rotem, Mor Moskovitz, Ari Raphael

**Affiliations:** a Davidoff Cancer Center, Rabin Medical Center-Beilinson Hospital, Petah Tikva, Israel; b Samueli Integrative Cancer Pioneering Institute, Beilinson Hospital, Petah Tikva, Israel; c Gray Faculty of Medical and Health Sciences, Tel Aviv University, Tel Aviv, Israel

**Keywords:** Small cell lung cancer, Chemoimmunotherapy, Platinum, Meta-analysis

## Abstract

**Introduction:**

SCLC is an aggressive malignancy for which platinum-based chemotherapy is the cornerstone of treatment. Although immunotherapy has redefined the standard of care, the optimal choice between cisplatin and carboplatin remains debated. This study evaluated the impact of platinum backbone selection on survival outcomes across the SCLC spectrum in the immunotherapy era.

**Methods:**

A systematic review and meta-analysis were performed on randomized controlled trials evaluating first-line chemoimmunotherapy with platinum-based regimens in SCLC. Outcomes were stratified by disease stage and platinum backbone (carboplatin versus cisplatin). Survival end points included progression-free survival (PFS) and overall survival (OS).

**Results:**

Seven trials (3059 patients) were analyzed. In extensive-stage SCLC (*n* = 1843), both backbones provided comparable benefits when combined with immunotherapy; the pooled OS hazard ratio (HR) for cisplatin was 0.71 (95% confidence interval [CI]: 0.57–0.89; *p* = 0.003) and 0.76 for carboplatin (95% CI: 0.68–0.85; *p* < 0.001). In limited-stage SCLC (*n* = 1216), the addition of immunotherapy improved OS (HR: 0.71; 95% CI: 0.54–0.92; *p* = 0.01) and PFS (HR: 0.70; 95% CI: 0.56–0.87; *p* = 0.002) in carboplatin-treated patients, whereas, in cisplatin-treated patients the benefit did not reach statistical significance for OS (HR: 1.15; 95% CI: 0.77–1.72; *p* = 0.49) or PFS (HR: 1.02; 95% CI: 0.81–1.28; *p* = 0.87).

**Conclusion:**

These findings invite a reconsideration of the platinum hierarchy in SCLC management. By demonstrating that carboplatin provides a more effective backbone in the limited-stage setting and comparable efficacy in the extensive-stage setting, this analysis suggests that the historical prioritization of cisplatin may no longer be justified in the era of immunotherapy. Prioritizing carboplatin as a preferred agent may optimize tolerability while preserving or improving survival outcomes across the SCLC spectrum.

## Introduction

The treatment landscape for SCLC has shifted with the advent of immunotherapy. The addition of PD-(L)1 inhibitors to platinum-etoposide chemotherapy has improved survival in both limited-stage (LS-SCLC) and extensive-stage disease (ES-SCLC).^1^, ^2^, ^3^ Traditionally, concurrent chemoradiation is recommended for the management of LS-SCLC followed by maintenance immunotherapy, and chemoimmunotherapy is recommended for ES-SCLC. Regardless of stage, a platinum backbone of either cisplatin or carboplatin remains the standard of care.^4^, ^5^, ^6^

Cisplatin is associated with significant nonhematological toxicities, including nephrotoxicity, neurotoxicity, and ototoxicity.^7^^,^^8^ Carboplatin offers a more favorable toxicity profile but has historically been viewed as potentially inferior in efficacy, particularly in the curative-intent setting of LS-SCLC.^4^, ^5^, ^6^ Despite four decades of platinum-based therapy in SCLC, the optimal choice between cisplatin and carboplatin remains a subject of debate.^7^^,^^9^ Critically, this comparison has not been rigorously re-evaluated after the incorporation of immune checkpoint inhibitors (ICIs) into frontline regimens. To address this gap, we conducted a systematic review and meta-analysis of trials that compared carboplatin-etoposide with cisplatin-etoposide as first-line therapy in SCLC to evaluate survival outcomes, stratified by disease stage.

## Methods

### Search Strategy and Selection Criteria

A systematic literature search of the PubMed/MEDLINE database was performed on January 5, 2026. The search strategy included the terms “small cell lung cancer” and “atezolizumab or durvalumab or avelumab or cosibelimab or nivolumab or pembrolizumab or tislelizumab or dostarlimab or cemiplimab or toripalimab or retifanlimab or penpulimab” and “chemotherapy or platinum” (Supplementary materials). Supplemental searches were performed across major oncology meetings to identify updated abstracts and presentations, specifically the World Conference on Lung Cancer, the American Society of Clinical Oncology, the European Society for Medical Oncology, and the European Lung Cancer Congress. For trials with multiple records, only the most updated results were included. The criteria for inclusion in the meta-analysis were as follows: (1) studies enrolling adult patients with SCLC; (2) phase 2 or phase 3 randomized controlled trials (RCTs) evaluating first-line platinum-based doublets in combination with, or followed by, any anti–PD-(L)1 with Food and Drug Administration approval (regardless of indication); and (3) reported progression-free survival (PFS) or overall survival (OS) stratified by the specific platinum agent used.

As this meta-analysis used only published, deidentified data from completed RCTs, institutional review board approval and individual informed consent were not required.

### Data Analysis

Variables extracted from each study included trial identification, study design, and sample sizes per arm. We specifically recorded the number of patients and events for each platinum-based combination, including the median survival and hazard ratios (HRs) with 95% confidence intervals (CIs) for both outcomes, stratified by the use of cisplatin or carboplatin. The main objectives were as follows: (1) to estimate the pooled summary HR of PFS and OS for each platinum-based subgroup and (2) to compare the survival outcomes for carboplatin- versus cisplatin-based chemoimmunotherapy combinations.

Restricted maximum likelihood random-effects models were used to pool effect sizes, and Cochran’s Q test and the *I*^2^ statistic were used to quantify heterogeneity; the same Q-based framework was used to test for treatment-by-platinum and treatment-by-stage interactions. The strength of associations was quantified by the magnitude of HRs and 95% CIs. To pool survival outcomes, individual patient data (IPD) were reconstructed from published Kaplan-Meier curves. When subgroup-specific curves were unavailable, subpopulation data were simulated using a genetic algorithm (detailed in the Supplementary Materials). Control arm outcomes were also pooled with stage-stratified estimates from the COCIS meta-analysis^8^ to isolate platinum effects independent of immunotherapy. Tests were two-sided, and a *p* value less than 0.05 was considered statistically significant.

## Results

Of 537 identified records, seven trials were included in the meta-analysis (Fig. 1)^1^, ^2^, ^3^^,^^10^, ^11^, ^12^, ^13^: four in the extensive-stage setting (CASPIAN, IMpower-133, KEYNOTE-604, and RATIONALE-312 trials)^2^^,^^3^^,^^11^^,^^12^ and three in the limited-stage setting (ADRIATIC, ACHILES, and NRG-LU005 trials).^1^^,^^10^^,^^13^ In the limited-stage trials, ICI was administered sequentially as maintenance therapy after chemoradiotherapy in the ADRIATIC and ACHILES protocols, whereas it was initiated concurrently with chemoradiotherapy in the NRG-LU005 trial. A total of 3059 patients were included, with 1843 in the ES-SCLC cohort (80% carboplatin and 20% cisplatin) and 1216 in the LS-SCLC cohort (40% carboplatin and 60% cisplatin). Supplementary Table 1 summarizes the characteristics of the selected trials. The median follow-up ranged from 22.9 and 45.1 months.

**Figure 1:**
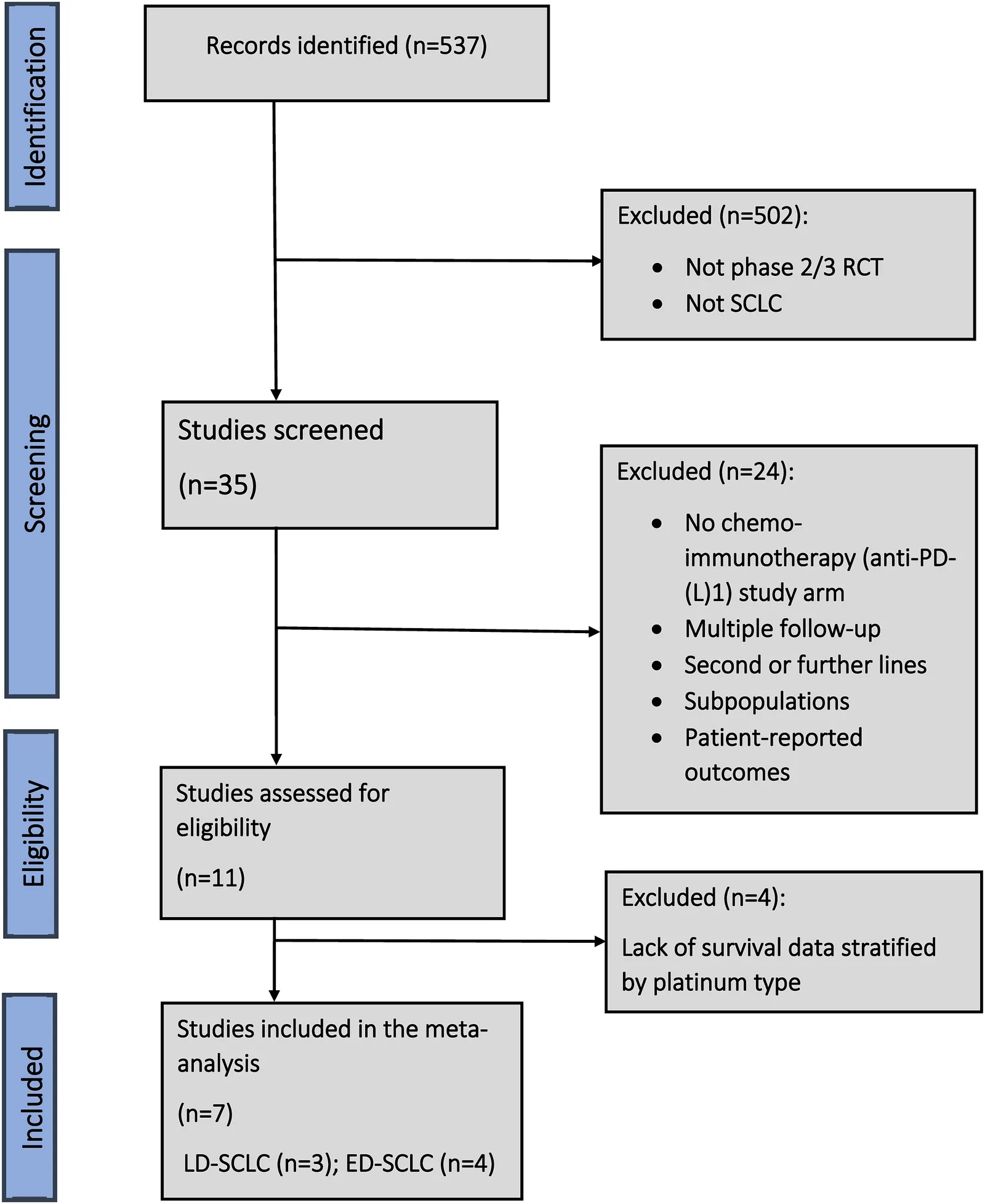
The PRISMA flow diagram detailing the screening and identification of eligible literature. ES-SCLC, extensive-stage SCLC; LS-SCLC, limited-stage SCLC; PRISMA, Preferred Reporting Items for Systematic Reviews and Meta-Analyses; RCT, randomized controlled trial.

### Efficacy: PFS and OS

The analysis of PFS revealed diverging outcomes based on the choice of platinum backbone and disease stage (Fig. 2). In patients receiving a cisplatin-based regimen, the pooled analysis demonstrated a statistically significant PFS benefit from ICI combination in the ES-SCLC cohort (HR = 0.66; 95% CI: 0.49–0.88; *I*^2^ = 0.0%). Conversely, no significant PFS improvement was observed in the LS-SCLC subgroup treated with cisplatin (HR = 1.02; 95% CI: 0.81–1.28; *I*^2^ = 27.2%). In contrast, the carboplatin backbone yielded consistent results across both disease stages. In the ES-SCLC cohort, the pooled HR for PFS was 0.71 (95% CI: 0.61–0.83; *I*^2^ = 20.5%). A similar significant benefit was maintained in the LS-SCLC setting (HR = 0.70; 95% CI: 0.56–0.87; *I*^2^ = 0.0%). A treatment-by-platinum interaction test confirmed this effect modification in LS-SCLC (*p* = 0.021) but not in ES-SCLC (*p* = 0.63).

**Figure 2:**
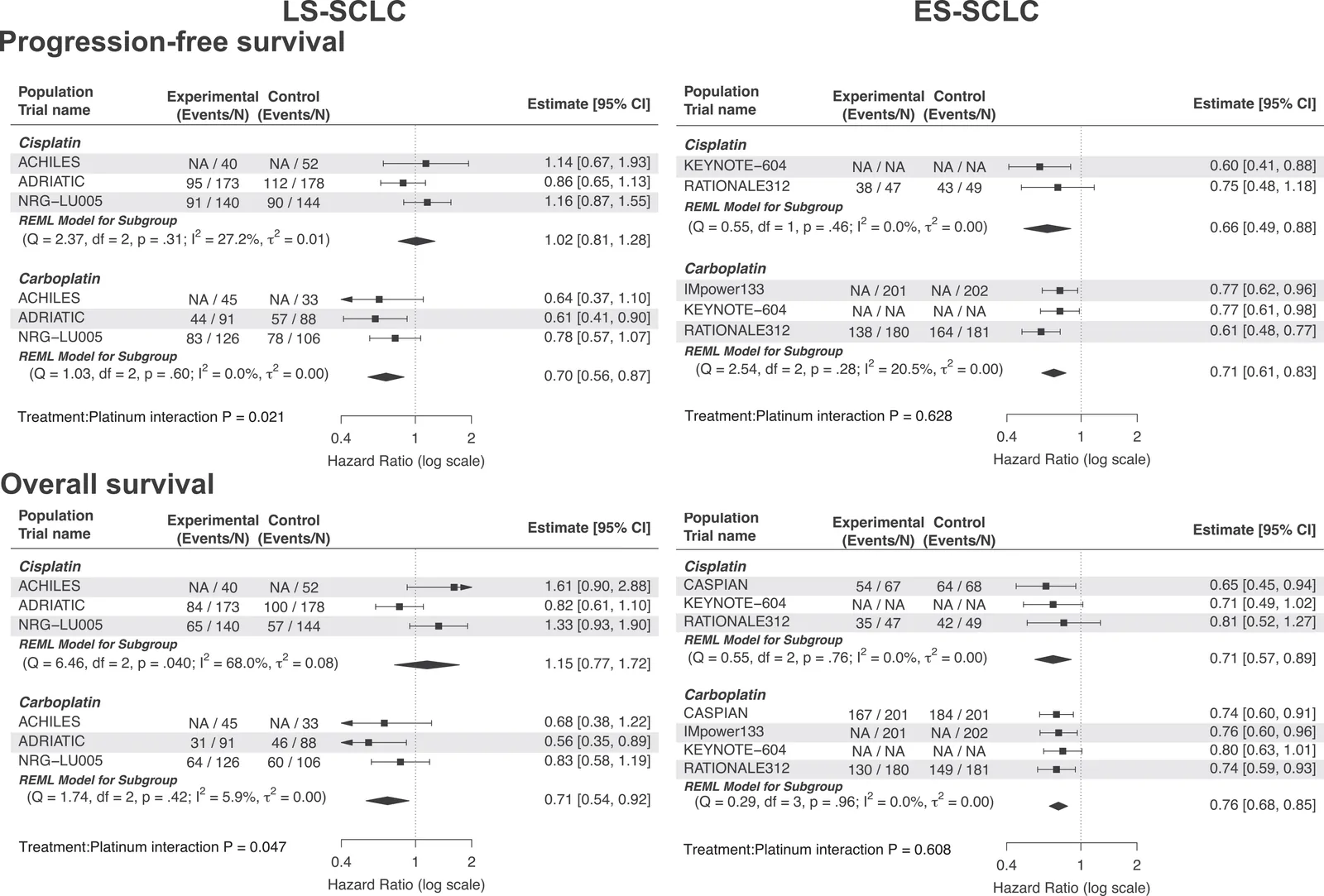
Forest plots of progression-free survival and overall survival stratified by SCLC stage and platinum agent. Forest plots show hazard ratios and 95% CIs for progression-free survival (top row) and overall survival (bottom row), stratified by limited-stage (left) versus extensive-stage disease (right). Studies are further subgrouped by the use of cisplatin or carboplatin. Diamonds represent pooled estimates. Hazard ratios less than 1 favor the experimental treatment. CI, confidence interval; ES-SCLC, extensive-stage SCLC; LS-SCLC, limited-stage SCLC; NA, not applicable.

The OS findings were consistent with the PFS analysis (Fig. 2). For patients treated with a cisplatin backbone, a significant OS benefit from ICI combination was identified in the ES-SCLC group (HR = 0.71; 95% CI: 0.57–0.89; *I*^2^ = 0.0%). However, the pooled OS analysis for LS-SCLC patients receiving cisplatin did not reach statistical significance (HR = 1.15; 95% CI: 0.77–1.72; *I*^2^ = 68.0%). In the carboplatin-treated cohorts, survival benefits were robust and consistent regardless of stage. The pooled OS HR was 0.76 for ES-SCLC (95% CI: 0.68–0.85; *I*^2^ = 0.0%) and 0.71 for LS-SCLC (95% CI: 0.54–0.92; *I*^2^ = 5.9%). As with PFS, the treatment-by-platinum interaction was significant for OS in LS-SCLC (*p* = 0.047) but not in ES-SCLC (*p* = 0.61).

Analyses based on reconstructed IPD (Fig. 3, Supplementary Fig. 1) produced results consistent with the meta-analysis. Furthermore, the worse OS in the LS-SCLC carboplatin control group, contrasting with the ES-SCLC findings, suggests that this cohort comprised patients with lower baseline fitness (Supplementary Figs. 2 and 3). In the absence of immunotherapy, cisplatin and carboplatin did not differ significantly in terms of PFS or OS in either stage, and no treatment-by-stage interaction was observed when control-arm outcomes were pooled with the COCIS meta-analysis (Supplementary Fig. 7). Sensitivity analyses without NRG-LU005, which used concurrent chemoimmunotherapy, are found in Supplementary Figures 4 to 6.

**Figure 3:**
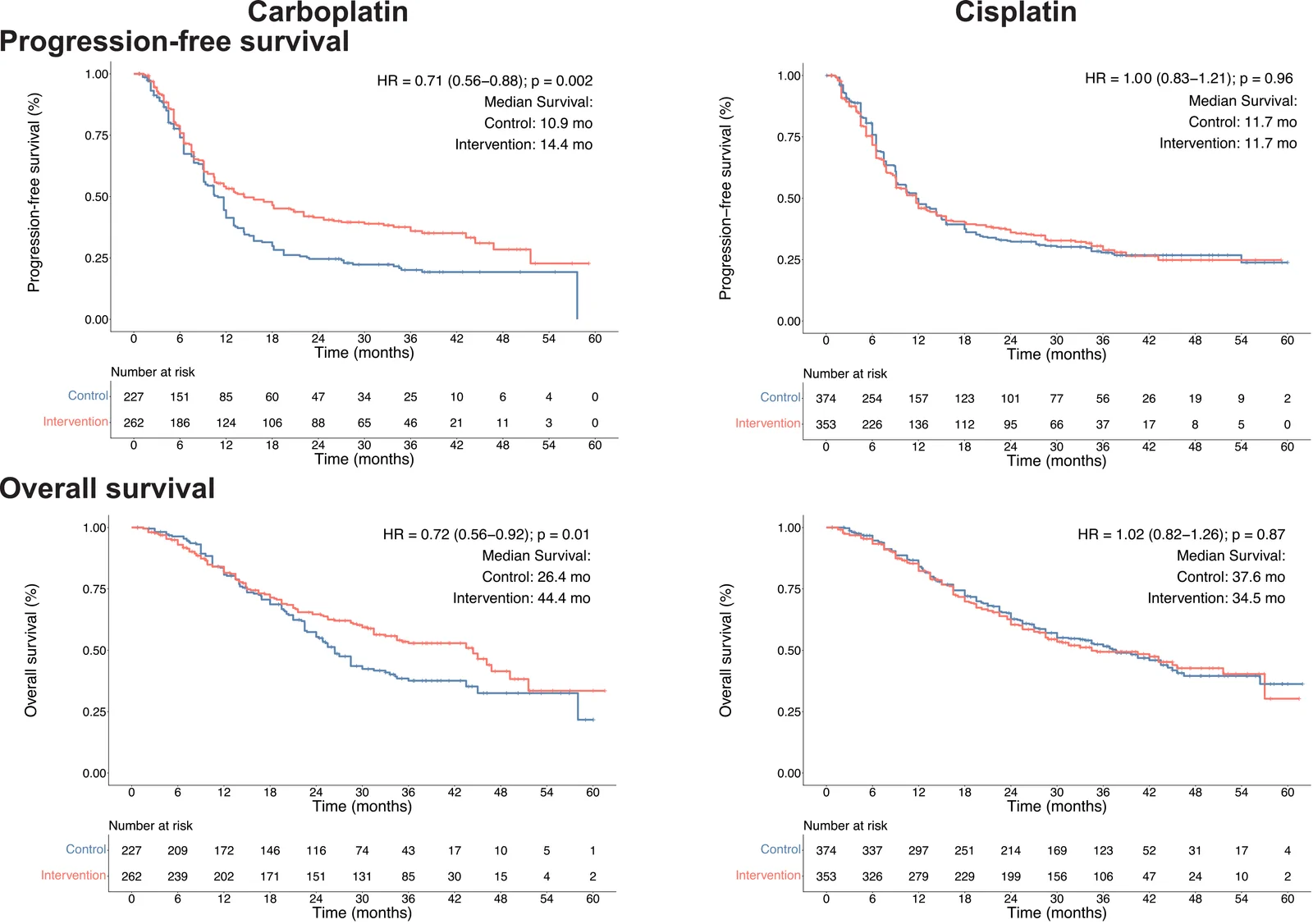
Pooled individual patient-level data survival analysis for limited-stage SCLC. Kaplan-Meier curves demonstrate progression-free survival (top row) and overall survival (bottom row) stratified by the use of carboplatin (left) or cisplatin (right). To generate pooled outcomes, the analysis integrates directly reconstructed individual patient-level data from available published graphs with algorithm-simulated subpopulation data for trials where specific platinum-stratified curves were absent. Red and blue curves denote the experimental and control arms, respectively. CI, confidence interval; HR, hazard ratio.

## Discussion

This meta-analysis highlights a notable divergence in the efficacy of platinum backbones when they are combined with immunotherapy in SCLC. In the extensive-stage setting, both cisplatin- and carboplatin-based regimens provide comparable and statistically significant survival benefits. However, in the limited-stage setting, carboplatin yielded a significant survival advantage, whereas cisplatin failed to reach statistical significance for either survival end point.

These divergent results in limited-stage disease have significant implications for clinical practice and future guideline development. Traditionally, cisplatin has been the preferred platinum agent for fit patients with limited-stage disease, with carboplatin reserved for patients for whom cisplatin is contraindicated or not tolerated.^4^, ^5^, ^6^ Current guidelines largely reflect expert consensus and historical practice, despite evidence, including the COCIS meta-analysis, showing no survival advantage for cisplatin over carboplatin in LS- or ES-SCLC.^7^, ^8^, ^9^^,^^14^ However, COCIS was limited by small study numbers, mixed disease stages, and heterogeneous chemotherapy regimens.^8^ To our knowledge, this meta-analysis is the first to strictly evaluate stage-stratified, platinum-etoposide regimens. Our findings suggest that cisplatin offers no therapeutic advantage over carboplatin, and, in the limited-stage setting, a detrimental effect cannot be excluded. The variability in outcomes may stem from the distinct toxicological profiles of these agents when combined with thoracic radiation and subsequent maintenance therapy. Cisplatin is associated with a higher incidence of nonhematological toxicities, which can negatively affect treatment adherence and long-term tolerability. Within the curative-intent setting, the use of the less toxic carboplatin may facilitate more timely and complete delivery of concurrent chemoradiation, despite a higher risk of myelosuppression.^9^ These toxicities may also persist beyond chemoradiation and compromise delivery of the subsequent maintenance immunotherapy. A complementary mechanism may be immunologic rather than logistic. The thymus lies within or immediately adjacent to mediastinal treatment volumes, and incidental thymic irradiation has recently been associated with impaired thymic function and a higher risk of distant metastasis in patients with NSCLC treated with chemoradiotherapy, most markedly in those who received consolidation immunotherapy.^15^ Under this hypothesis, if cisplatin, as the more potent radiosensitizer, accentuates this incidental thymic injury, it may impair the development of a robust T-cell-mediated antitumor immune response, thereby undermining the efficacy of subsequent PD-(L)1 blockade. Such a mechanism would operate only where thoracic radiation is delivered, consistent with the divergence being confined to limited-stage disease. Because maintenance is administered only in the experimental arm, such an effect may attenuate the immunotherapy benefit specifically in cisplatin-treated patients, while leaving the control comparison unaffected. The absence of a corresponding platinum difference in the pooled control arm and COCIS comparison does not establish equivalence, because platinum is selected rather than randomized, and carboplatin is preferentially assigned to less-fit patients, biasing that comparison against it. That the carboplatin advantage nonetheless emerged once immunotherapy was added, despite this cohort's lower baseline fitness, suggests that the benefit is, if anything, underestimated. Conversely, the consistent survival benefits observed with carboplatin backbones in both limited and extensive stages suggest that carboplatin provides a robust and predictable backbone. Even in the extensive-stage setting, where efficacy appears comparable, the more favorable toxicity profile of carboplatin often makes it the clinically preferable choice. Furthermore, the failure of cisplatin-based cohorts to reach statistical significance in the limited-stage setting warrants a re-evaluation of the “cisplatin-first” dogma. If immunotherapy provides more substantial survival gains when paired with a more tolerable carboplatin backbone, the clinical rationale for strictly choosing cisplatin weakens. These data suggest that carboplatin is not merely an alternative for frail patients but a preferred backbone across the entire spectrum of SCLC.

Several limitations should be considered. Data were derived from subgroup analyses of RCTs rather than head-to-head comparisons of cisplatin and carboplatin and are therefore susceptible to baseline imbalance. In particular, the carboplatin-treated cohorts included patients with lower baseline fitness, potentially leading to an underestimation of carboplatin's efficacy relative to cisplatin. Survival outcomes were reconstructed from published Kaplan-Meier curves, and where subgroup-specific curves were not reported, the underlying data were derived indirectly, which may introduce additional uncertainty. The number of eligible trials was limited in both stages, and some of the limited-stage cisplatin analyses reported statistical heterogeneity. The regimens were also not uniform across trials, differing in the timing of immunotherapy relative to chemoradiation, and in one trial (ACHILES), patients did not consistently receive the same platinum agent across cycles. The sample size for cisplatin-treated patients in the ES-SCLC cohort was relatively small. Information regarding platinum-specific toxicity and treatment completion rates was not readily available in the analyzed reports. Despite these limitations, by restricting inclusion to trials reporting platinum-stratified outcomes, this study provides a comprehensive stage-specific evaluation of this clinical question.

In conclusion, our findings support a paradigm shift that challenges a decades-old clinical hierarchy in the management of SCLC. By demonstrating that a carboplatin-based regimen provides a more effective backbone in the limited-stage setting and comparable outcomes in the extensive-stage setting, this analysis argues that the historical prioritization of cisplatin may no longer be justified. The evidence indicates that the contemporary standard of care should transition toward a more patient-centered approach, where carboplatin is recognized not merely as an alternative but as a preferred agent for maximizing tolerability and the survival benefits offered by immunotherapy. Redefining this treatment paradigm is essential to optimize patient outcomes and ensure that recent clinical advances are fully realized across all stages of the disease.

## CRediT Authorship Contribution Statement

**Tomer Meirson:** Conceptualization, Methodology, Formal analysis, Data curation, Project administration, Writing - original draft, Writing - review & editing.

**Hadas Ditzian Kugler:** Data curation, Formal analysis, Writing - review & editing.

**Ekaterina Hanovich:** Writing - review & editing.

**Ofer Rotem:** Writing - review & editing.

**Mor Moskovitz:** Supervision, Writing - review & editing.

**Ari Raphael:** Supervision, Data curation, Writing - review & editing.

## Disclosure

Dr. Meirson reports receiving personal fees from Purple Biotech and a research grant from Roche. Dr. Rotem reports being a speaker for AstraZeneca, Boehringer Ingelheim, BMS (Bristol Myers Squibb), MSD (Merck Sharp & Dohme), Novartis, Pfizer, Roche, Takeda, and Teva, and being a consultant for Rhenium, NucleaiMD, and Edocate. Dr. Moskovitz reports receiving a research grant from AstraZeneca; being a speaker for AstraZeneca, Roche, MSD, BMS, Pfizer, Amgen, AbbVie, J&J (Johnson & Johnson), and Takeda; and being a consultant for MSD, J&J, Bayer, Amgen, and Takeda. Dr. Raphael reports receiving a research grant from MSD and Roche; being a speaker for AstraZeneca, BMS, MSD, Pfizer, Roche, and Takeda; and being a consultant for Rhenium and Boehringer Ingelheim. The remaining authors declare no conflict of interest.

## Footnotes

Note: To access the supplementary material accompanying this article, visit the online version of the *JTO Clinical and Research Reports* at www.jtocrr.org and at https://doi.org/10.1016/j.jtocrr.2026.101055.
