# Supplementary material for: Rethinking the Platinum Standard in SCLC: A Meta-Analysis of Limited- and Extensive-Stage Disease in the Immunotherapy Era
Source: JTO Clin Res Rep. 2026 Jul 24;7(11):101055. doi: 10.1016/j.jtocrr.2026.101055 (PMC13675750; doi:10.1016/j.jtocrr.2026.101055)
Supplement: Supplementary file 1 [file mmc1.docx]

**SUPPLEMENTARY MATERIALS**

**Study identification**

The query used for the identification of eligible studies in the PubMed/MEDLINE database was as follows:

("small cell lung carcinoma"[MeSH Terms] OR (("small cell lung cancer"[ti] OR "SCLC"[ti]) NOT "non-small"[ti])) AND (atezolizumab OR durvalumab OR avelumab OR cosibelimab OR nivolumab OR pembrolizumab OR tislelizumab OR dostarlimab OR cemiplimab OR toripalimab OR retifanlimab OR penpulimab) AND (chemotherapy OR platinum OR cisplatin OR carboplatin OR etoposide)

**Data Pooling Strategy for Survival Outcomes**

To pool progression-free survival (PFS) and overall survival (OS) across the included trials, a hierarchical approach to data extraction was employed depending on data availability (summarized in Supplementary Table 2). Whenever Kaplan-Meier survival curves for specific platinum subgroups (carboplatin or cisplatin) were available in the published literature, individual patient-level data (IPD) were directly reconstructed. Conversely, for trials that reported survival metrics for the target subgroups but lacked published Kaplan-Meier curves, we utilized a mathematical algorithm to simulate and isolate the required subpopulation data from the overall intention-to-treat cohort.

**Survival Data Reconstruction**

When published Kaplan-Meier curves were available, IPD were reconstructed using a two-stage process. First, the survival curves were digitized with WebPlotDigitizer to extract raw coordinates of time and survival probability. Second, these data were processed using the iterative algorithm proposed by Guyot et al. to reconstruct individual patient-level records. To ensure accuracy, the reconstruction incorporated reported number-at-risk tables to synchronize the data with the original trial population. The fidelity of the reconstructed data was validated by comparing the derived hazard ratio with the originally reported value. For the non-immunotherapy comparator analysis (Supplementary Figure 7), stage-stratified cisplatin-versus-carboplatin hazard ratios and 95% confidence intervals were extracted from the published forest plots of the COCIS meta-analysis using WebPlotDigitizer and entered as aggregate effect sizes, with the direction of effect inverted to match the carboplatin-versus-cisplatin orientation used throughout.

**Subpopulation Simulation Algorithm**

To identify the patient subset matching the published carboplatin or cisplatin subpopulation when specific curves were unavailable, we utilized a real-valued genetic algorithm (GA) to optimize the selection of patients from the intention-to-treat population. To strictly enforce the reported sample sizes and event counts, the cohort was stratified by treatment arm and event status. In each GA iteration, patients were assigned continuous priority scores (0–1), and the exact number of top-scoring patients required for each stratum was selected.

**Optimization and Constraints**

Candidate subpopulations were evaluated using a Cox proportional hazards model. The GA minimized a cost function defined by the sum of squared normalized errors between the simulated and target hazard ratio (HR) and 95% confidence intervals (CI), with the HR error weighted tenfold.

To prevent biologically implausible survival curve braiding, a geometric constraint was applied. Survival probabilities were evaluated at 10 evenly spaced time points; if the experimental arm dropped >5% below the control arm, a squared penalty proportional to the crossing depth was added to the cost function.

Analyses were performed using the R packages GA and survival. The algorithm ran with a population size of 100, mutation rate of 0.2, and a 2,000-generation maximum, utilizing an early stopping trigger of 50 consecutive generations without improvement. All analyses were performed with the R Statistical Software v4.5.2.

**Supplementary Table 1.** Study design and characteristics

| **Trial** | **Stage** | **Phase** | **ICI timing** | **Study Years (Enrollment / Publicat.)** | **N (ITT)** | **Platinum Backbone (N/%)** | **Median Follow-up (months)** |
| --- | --- | --- | --- | --- | --- | --- | --- |
| **LS-SCLC** |  |  |  |  |  |  |  |
| **ADRIATIC** | LS | 3 | Maintenance | 2018–2024 / 2024 | 530 | **Cis:** 351 (66%) / **Carbo:** 179 (34%) | 37.2 |
| **NRG-LU005** | LS | 2/3 | Concurrent + maintenance | 2019–2023 / 2026 | 544 | **Cis:** 322 (59%) / **Carbo:** 222 (41%) | 23.8 |
| **ACHILES** | LS | 2 | Maintenance | 2018–2024 / 2025 | 170 | **Cis only:** 92 (54%) /  **Carbo:** 78 (46%) | 45.1 |
| **ES-SCLC** |  |  |  |  |  |  |  |
| **CASPIAN** | ES | 3 | Concurrent | 2017–2018 / 2019 | 537 | **Cis:** 135 (25%) / **Carbo:** 402 (75%) | 39.4 |
| **IMpower-133** | ES | 3 | Concurrent | 2016–2018 / 2018 | 403 | **Carbo:** 403 (100%) | 22.9 |
| **KEYNOTE-604** | ES | 3 | Concurrent | 2017–2019 / 2020 | 453 | **Cis:** 105 (23%) / **Carbo:** 348 (77%) | 43.3 |
| **RATIONALE-312** | ES | 3 | Concurrent | 2019–2021 / 2024 | 457 | **Cis:** 96 (21%) / **Carbo:** 361 (79%) | 39.8 |
| ICI, immune checkpoint inhibitor; LS-SCLC, limited-stage small cell lung cancer; ES-SCLC, extensive-stage small cell lung cancer; Cis, cisplatin; Carbo, carboplatin; N (ITT), number of patients in the intent-to-treat population. | | | | | | | |

**Supplementary Table 2.** Data extraction methodology for pooling progression-free and overall survival outcomes.

| **Trial** | **PFS** | **OS** |
| --- | --- | --- |
| **LS-SCLC** | | |
| LU005 | Simulated | Simulated |
| ADRIATIC | Simulated | Reconstructed |
| ACHILES | Reconstructed | Reconstructed |
| **ES-SCLC** | | |
| RATIONALE-312 | Simulated | Simulated |
| KEYNOTE-604 | Missing* | Missing* |
| IMpower133 | Reconstructed^†^ | Reconstructed^†^ |
| CASPIAN | Missing^∥^ | Reconstructed |
| PFS – Progression-free survival; OS – overall survival. SCLC – small cell lung cancer.  LS-SCLC – limited stage SCLC. ES-SCLC – extensive stage SCLC.  Reconstructed – refers to individual patient-level data (IPD) derived directly from published Kaplan-Meier curves using the Guyot et al. method.  Simulated – refers to subpopulation data derived via the genetic algorithm.  * Missing event data stratified by platinum-basted treatment.  † The study included only carboplatin-based regimens.  ∥ No published PFS results. | | |

**Supplementary Figure 1. Survival outcomes comparing platinum backbones within treatment arms for limited-stage SCLC (LS-SCLC).** Kaplan-Meier curves demonstrate progression-free survival (top row) and overall survival (bottom row) for patients assigned to the experimental arm (left) or the control arm (right). To generate pooled outcomes, the analysis integrates directly reconstructed IPD from available published graphs with algorithm-simulated subpopulation data for trials where specific platinum-stratified curves were absent. Red curves denote patients treated with a carboplatin backbone, and blue curves denote patients treated with a cisplatin backbone. HR, hazard ratio; CI, confidence interval.

**Supplementary Figure 2. Pooled individual patient-level data (IPD) survival analysis for extensive-stage SCLC (ES-SCLC).** Kaplan-Meier curves demonstrate progression-free survival (top row) and overall survival (bottom row) stratified by the use of carboplatin (left) or cisplatin (right). To generate pooled outcomes, the analysis integrates directly reconstructed IPD from available published graphs with algorithm-simulated subpopulation data for trials where specific platinum-stratified curves were absent. Red and blue curves denote the experimental and control arm, respectively. HR, hazard ratio; CI, confidence interval.

**Supplementary Figure 3. Survival outcomes comparing platinum backbones within treatment arms for extensive-stage SCLC (ES-SCLC).** Kaplan-Meier curves demonstrate progression-free survival (top row) and overall survival (bottom row) for patients assigned to the experimental arm (left) or the control arm (right). To generate pooled outcomes, the analysis integrates directly reconstructed IPD from available published graphs with algorithm-simulated subpopulation data for trials where specific platinum-stratified curves were absent. Red curves denote patients treated with a carboplatin backbone, and blue curves denote patients treated with a cisplatin backbone. HR, hazard ratio; CI, confidence interval.

**Supplementary Figure 4. Forest plots of progression-free survival (PFS) and overall survival (OS) for LS-SCLC excluding NRG-LU005 stratifiedf by platinum agent.** Forest plots show hazard ratios (HR) and 95% confidence intervals (CIs) for PFS (top row) and OS (bottom row), stratified by limited-stage (left) versus extensive-stage disease (right). Studies are further subgrouped by the use of cisplatin or carboplatin. Diamonds represent pooled estimates. HR < 1 favors the experimental treatment. SCLC, limited-disease small cell lung cancer; LS-SCLC, limited-stage SCLC; ES-SCLC, extensive stage SCLC

**Supplementary Figure 5. Pooled individual patient-level data (IPD) survival analysis for limited-stage SCLC (LS-SCLC) excluding NRG-LU005.** Kaplan-Meier curves demonstrate progression-free survival (top row) and overall survival (bottom row) stratified by the use of carboplatin (left) or cisplatin (right). To generate pooled outcomes, the analysis integrates directly reconstructed IPD from available published graphs with algorithm-simulated subpopulation data for trials where specific platinum-stratified curves were absent. Red and blue curves denote the experimental and control arm, respectively. HR, hazard ratio; CI, confidence interval.

**Supplementary Figure 6. Survival outcomes comparing platinum backbones within treatment arms for limited-stage SCLC (LS-SCLC) excluding NRG-LU005.** Kaplan-Meier curves demonstrate progression-free survival (top row) and overall survival (bottom row) for patients assigned to the experimental arm (left) or the control arm (right). To generate pooled outcomes, the analysis integrates directly reconstructed IPD from available published graphs with algorithm-simulated subpopulation data for trials where specific platinum-stratified curves were absent. Red curves denote patients treated with a carboplatin backbone, and blue curves denote patients treated with a cisplatin backbone. HR, hazard ratio; CI, confidence interval.

**Supplementary Figure 7. Forest plots of the platinum comparison in the absence of immunotherapy, stratified by SCLC stage**. Forest plots show hazard ratios (HR) and 95% confidence intervals (CIs) for progression-free survival (top) and overall survival (bottom), comparing carboplatin with cisplatin in the control (non immunotherapy) arms of the included trials pooled with the stage-stratified estimates of the COCIS meta-analysis, subgrouped by limited-stage versus extensive-stage disease. Diamonds represent pooled estimates. The treatment-by-stage interaction P value is shown for each endpoint. HR < 1 favors carboplatin. SCLC, small cell lung cancer; LS-SCLC, limited-stage SCLC; ES-SCLC, extensive stage SCLC.
